# Erratum: Moreira, J., et al., Spin-Coated Polysaccharide-Based Multilayered Freestanding Films with Adhesive and Bioactive Moieties. *Molecules* 2020, *25*, 840

**DOI:** 10.3390/molecules25122727

**Published:** 2020-06-12

**Authors:** Joana Moreira, Ana C. Vale, Ricardo A. Pires, Gabriela Botelho, Rui L. Reis, Natália M. Alves

**Affiliations:** 1 3Bs Research Group, I3Bs—Research Institute on Biomaterials, Biodegradables and Biomimetics, University of Minho, Headquarters of the European Institute of Excellence on Tissue Engineering and Regenerative Medicine, Avepark, Barco, 4805-017 Guimarães, Portugal; joana.lagoa@hotmail.com (J.M.); rpires@i3bs.uminho.pt (R.A.P.); rgreis@i3bs.uminho.pt (R.L.R.); 2 ICVS/3B’s, Associate PT Government Laboratory, 4710-057 Braga/4805-017 Guimarães, Portugal; 3 The Discoveries Centre for Regenerative and Precision Medicine, Headquarters at University of Minho, Avepark, Barco, 4805-017 Guimarães, Portugal; 4 Department of Chemistry, University of Minho, Campus de Gualtar, 4710-057 Braga, Portugal; gbotelho@quimica.uminho.pt

The authors wish to make changes to the published paper [1]. 

## 2.1. UV-Vis Analysis of Catechol-Modified Polymers

In the original manuscript, there is a mistake concerning the word “Wavenumber” in the X-coordinate in Figure 1. The corrected word is “Wavelength”. The authors also wish to change mg•mL−1 to mg mL−1 in the legend of Figure 1; see corrected Figure 1 below.

## 2.6. Thermogravimetric Analysis (TGA)

There is a mistake with the word “CNT” in the legend of Figure 8. The corrected word is “CTR”; see corrected Figure 8 below.

## 5. Conclusions

The authors wish to change a sentence at the end of Section “Conclusions”. The original sentence is “These bioactive LbL freestanding films that combine good adhesion with improved mechanical properties could **find applications in the biomedical field, such as** guided hard tissue regeneration **(GTR) membranes**.” The corrected sentence is “These bioactive LbL freestanding films that combine good adhesion with improved mechanical properties could **be an alternative strategy** for guided hard tissue regeneration.”

Finally, there is a mistake with the word “cross-link” in the main text. The corrected word is “crosslink”. 

The authors apologize for any inconvenience caused and the change does not affect the scientific results. The manuscript will be updated, and the original will remain online on the article webpage at https://www.mdpi.com/1420-3049/25/4/840.

## Figures and Tables

**Figure 1:**
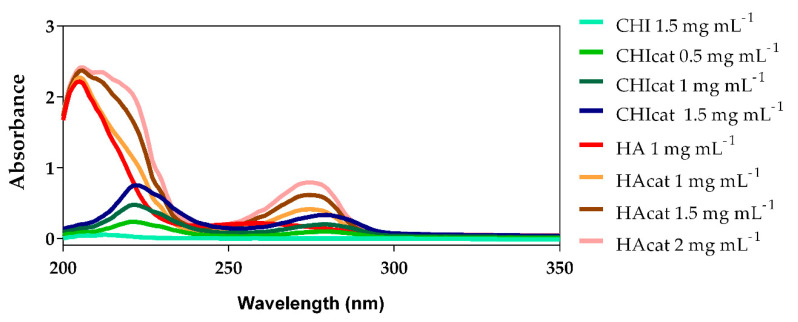
UV-Vis spectra of chitosan (CHI) and hyaluronic acid (HA), catechol-conjugated CHI (CHIcat) and catechol-conjugated HA (HAcat) (λ = 200–350 nm).

**Figure 8:**
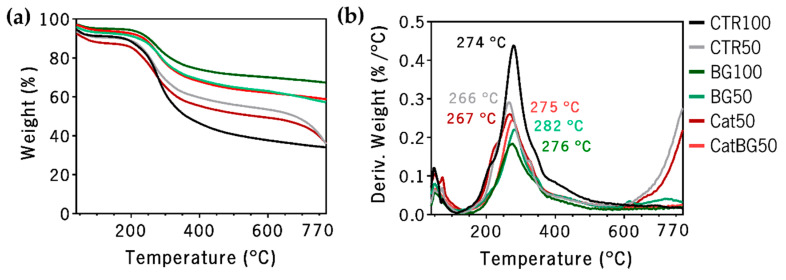
TGA thermograms of (**a**) weight loss and (**b**) derivative of the weight loss (DTGA) of the freestanding LbL films as a function of temperature.
